# Supplementary material for: Dual-Toxin-Producing Clostridium botulinum Strain Isolated from a Foodborne Botulism Case in Korea: Genomic and Functional Insights
Source: Toxins (Basel). 2025 Jun 12;17(6):299. doi: 10.3390/toxins17060299 (PMC12197393; doi:10.3390/toxins17060299)
Supplement: Supplementary file 1 [file toxins-17-00299-s001.zip › toxins-3668807-supplementary.pdf]

toxins

MDPI

Supplementary Material: Dual-Toxin-Producing *Clostridium botulinum* Strain Isolated from a Foodborne Botulism Case in Korea: Genomic and Functional Insights

Eun-Sun Choi, Chi-Hwan Choi, Jun-Ho Jeon, So-Hyeon Kim, Hyun-Ju Song, Hwajung Yi, Gi-eun Rhie and Yoon-Seok Chung

Table S1. Detailed information of *bont* genes used for phylogenetic analysis

Strain	Subtype	Accession number
Bf	B5	ABDP01000018
An436	B5	LFON01000008
CDC 4013	B5	GU271943
161.08	B5	KC471328
CDC69057	B5	QVAH01000158
★CB-2014001	B5	OP019001
657	B5	CP001081
CDC 3281	B5	GQ244313
PA9508B	B5/F2	CP102918
CDC 1758	B1	EF033127
okra	B1	CP000940
Hall 6517(B)	B1	EF028399
Osaka05	B6	AB302852
Okayama2011	B6	DAEWWX010000022
111	B2	AB084152
M-18/3	B2	FM865705
H151380082	B3	SGJT01000030
CDC 795	B3	EF028400
A2B3 87	B3	AUZB01000012
Surat Thani 2012(26898)	B8	KC714045
Maehongson 2010	B8	JQ964806
Bac-04-07755	B7	JQ354985
A2B7 92	B7	AUZA01000014
Eklund 17B	B4	EF051570
Templin	B4	MG545727
CDC706	B4	JX437192
161.08	F2	KC471329
An436	F2	LFON01000008
Bf	F2	ABDP01000023
CDC 4013	F2	GU213209
CDC69057	F2	QVAH01000105
CDC 3281	F2	Y13631

Copyright: © 2025 by the authors. Licensee MDPI, Basel, Switzerland. This article is an open access article distributed under the terms and conditions of the Creative Commons Attribution (CC BY) license (https://creativecommons.org/licenses/by/4.0/).

 https://doi.org/10.3390/toxins17060299

★CB-2014001	F2	OP019002
VPI 4257	F3	GU213227
CDC 54086	F3	GU213218
H078-01	F9	KX671959
CDC 54090	F5	GU213222
CDC 54085	F5	GU213217
CDC 54075	F5	GU213212
CDC 54079	F5	GU213215
CDC 54096	F5	GU213225
CDC 54074	F5	GU213211
CDC 54084	F5	GU213216
KA-173	F6	GU213230
VPI 2382	F6	GU213226
83F	F6	GU213229
VPI 7943	F6	GU213228
CDC 54089	F4	GU213221
CDC 54088	F4	GU213220
CDC 54087	F4	GU213219
CDC 54076	F4	GU213213
CDC 49930	F4	GU213210
CDC 54093	F4	GU213224
CDC 54091	F4	GU213223
CDC 54078	F4	GU213214
F 357	F8	AUZC01000009
SBPRU-0556	F8	JAAILF010000014
Langelang	F1	GU213203
4VI	F1	GU213205
12F	F1	GU213204
4VII	F1	GU213206
Pasteurized Crab	F1	GU213208
8g	F1	GU213207
CDC 59837	F7	GU213231
CDC 51192	F7	GU213232
CDC 35112	F7	GU213233
CDC 32356	F7	GU213234
CDC 51267	F7	GU213235

Table S2. Mouse lethality of CB-2014001 filtrates at 30°C and 37°C across dilution series.

**Condition**	**No. of mice alive/tested**	
	30°C	37 °C
**1:640**	0/2	0/2
**1:1280**	1/2	0/2
**1:2560**	2/2	0/2

**1:5120**	2/2	2/2
**1:10240**	2/2	2/2

Disclaimer/Publisher's Note: The statements, opinions and data contained in all publications are solely those of the individual author(s) and contributor(s) and not of MDPI and/or the editor(s). MDPI and/or the editor(s) disclaim responsibility for any injury to people or property resulting from any ideas, methods, instructions or products referred to in the content.
